# Multifunctional Inverse Opal‐Like TiO_2_ Electron Transport Layer for Efficient Hybrid Perovskite Solar Cells

**DOI:** 10.1002/advs.201500105

**Published:** 2015-06-17

**Authors:** Xiao Chen, Shuang Yang, Yi Chu Zheng, Ying Chen, Yu Hou, Xiao Hua Yang, Hua Gui Yang

**Affiliations:** ^1^ Key Laboratory for Ultrafine Materials of Ministry of EducationSchool of Materials Science and EngineeringEast China University of Science and Technology130 Meilong RoadShanghai200237China

**Keywords:** charge transporting, electron transport layer, inverse opal‐like structure, light harvesting efficiency, perovskite solar cell

## Abstract

**A novel multifunctional inverse opal‐like TiO_2_ electron transport layer (IOT‐ETL)** is designed to replace the traditional compact layer and mesoporous scaffold layer in perovskite solar cells (PSCs). Improved light harvesting efficiency and charge transporting performance in IOT‐ETL based PSCs yield high power conversion efficiency of 13.11%.

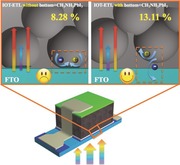

Organometallic halide perovskite based solar cells have recently attracted much attention due to their excellent photovoltaic performance, low‐cost, and low‐temperature processability.^1^, ^2^, ^3^ The first attempt was achieved by utilizing CH_3_NH_3_PbI_3_ as light harvester in dye‐sensitized solar cells based on liquid electrolyte and a power conversion efficiency (PCE) of 3.81% was obtained.^4^ However, further development of organometallic halide perovskite based solar cells was greatly inhibited by its instability in polar liquid electrolyte. Fortunately, replacing liquid electrolyte with solid‐state hole transport materials (HTM) boosted the PCE to 9.7%.^5^ During the past several years, the overall PCE of perovskite solar cells has increased observably. Recently, a PCE of 19.3% has been reported by Zhou et al., while the highest PCE of 20.1% has been confirmed by the national renewable energy laboratory.^6^

The structures of perovskite solar cells can be widely divided into two classes: mesoporous structure based perovskite solar cell and planar heterojunction based perovskite solar cell.^7^ For an efficient mesoporous structure based perovskite solar cell, the compact layer, mesoporous scaffold layer, perovskite light harvester, and HTM are essential elements.^8^ TiO_2_ and ZnO are typical materials for compact layer, which provide the electron transfer pathways and prevent the charge recombination between electrons and holes. Moreover, the TiO_2_ nanoparticles, TiO_2_ mesoporous structure, 1D TiO_2_ and ZnO have similar electron‐injecting mechanism and have been widely applied as mesoporous scaffold layers.^9^, ^10^, ^11^, ^12^, ^13^ When TiO_2_ or ZnO is utilized as both compact layer and mesoporous scaffold layer, these two layers could be named as electron transport layer (ETL). Moreover, enhanced light harvesting efficiency (LHE) is necessary for a good‐performance solar cell, while the LHE of conventional mesoporous structure TiO_2_ ETLs based perovskite solar cells has large potential to be improved.^14^ As a special structure with photonic band gap, the inverse opal photonic crystals have attracted much attention due to their ability in light manipulation in the past decade.^15^, ^16^ To the best of our knowledge, no research about inverse opal structures as ETL in perovskite solar cells has been reported so far. Furthermore, the traditional compact layer and scaffold layer in ETL are always prepared separately, which makes the fabrication of solar cells is time‐costing and expensive.^10^, ^11^, ^12^, ^13^ Attempts to simplify the manufacturing operation of ETL will be favorable for the large‐scale implementations of perovskite solar cells.

Here, we prepare a multifunctional inverse opal‐like TiO_2_ electron transport layer (IOT‐ETL) by a simple polystyrene‐assistant method for the first time, which combines the functions of the compact layer and mesoporous scaffold layer in perovskite solar cells. It demonstrates that the porous structure in IOT‐ETL could enhance the devices' LHE obviously. Moreover, the bottom of IOT‐ETL plays the same role as the traditional compact layer, transporting electrons and inhibiting the recombination of electrons and holes. As a result, the IOT‐ETL based perovskite solar cell yields a best PCE of 13.11%, higher than that of the conventional P25 mesoporous layer based perovskite solar cells (11.00%). To the best of our knowledge, this is the first study utilizing multifunctional inverse opal‐like TiO_2_ as ETL to obtain highly efficient perovskite solar cells with PCE >10%.

The fabrication procedure of IOT‐ETL based perovskite solar cells is shown in **Figure** **1**. In this procedure, the IOT‐ETL was prepared by a template‐assistant method. Two kinds of titanium precursor solution (Ti(SO_4_)_2_ aqueous solution and Ti(SO_4_)_2_ in PS emulsion) were spin‐coated on the etched FTO substrate respectively. After sintered at 773 K for 30 min, the template was removed and the porous structure was obtained. It is worth noting that a bottom of the IOT‐ETL film was fabricated by spin‐coating the Ti(SO_4_)_2_ aqueous solution. This bottom is significant for the improvement of the devices' overall PCE and will be discussed in the following text. CH_3_NH_3_PbI_3_ was prepared by a typical two‐step method, in which PbI_2_ was deposited on the IOT‐ETL films first followed by spin‐coating the CH_3_NH_3_I solution. After the spiro‐MeOTAD was deposited on the CH_3_NH_3_PbI_3_ film, Ag was thermally evaporated to form the back contact.

**Figure 1:**
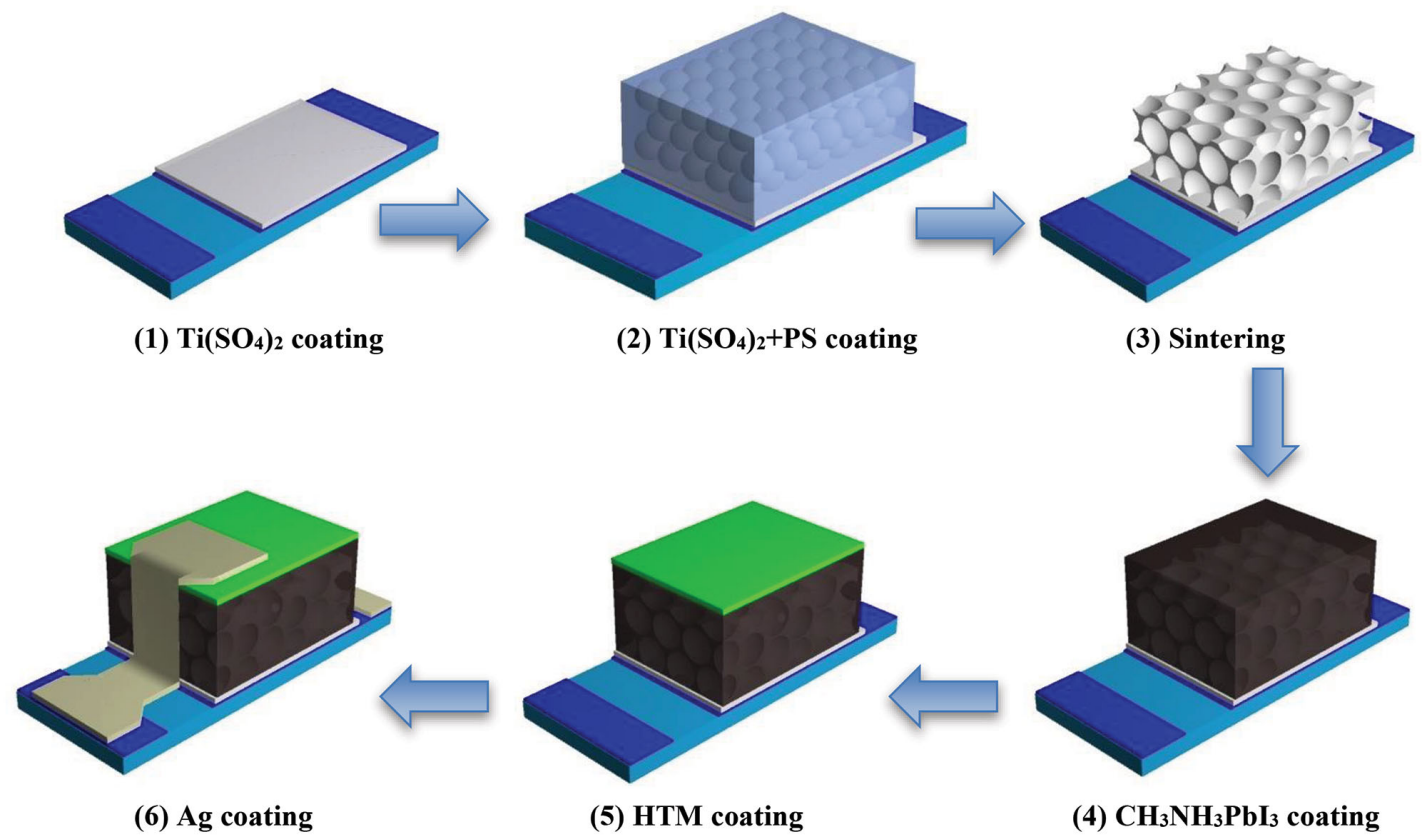
Fabrication procedure of the inverse opal‐like TiO_2_ electron transport layer based perovskite solar cells.

As shown in **Figure** **2**A, IOT‐ETL film is uniform and the diameter of the circular pore in IOT‐ETL film is about 100 nm, in consistent with that of synthesized polystyrene (PS) nanospheres (Figure S1, Supporting Information), indicating the porous structure could be derived well from the PS templates. From the X‐ray diffraction (XRD) pattern of the inverse opal‐like TiO_2_, all diffraction peaks match well with those of the anatase TiO_2_ (JCPDS No. 21–1272) (Figure S2A, Supporting Information). The inset image in Figure 2A indicates that the size of CH_3_NH_3_PbI_3_ cuboid above the IOT‐ETL film is between 100–150 nm, which is similar to the diameter of the circular pore. The larger pore size than that of conventional TiO_2_ mesoporous layer is beneficial for the filling of perovskite absorber.^17^ The thickness of the bare IOT‐ETL film prepared under 4000 rpm is around 150 nm (Figure 2B) and the inset image in Figure 2B demonstrates that the thickness of perovskite absorber is approximately 500 nm. This value is suitable for both light absorption and charge diffusion as the previous paper reported.^18^ TEM image (Figure 2C) shows that the nanopores are connected to each other by the TiO_2_ wall with a wall thickness of 6 nm. Moreover, conventional P25 mesoporous film with around 150 nm thickness was prepared by spin‐coating self‐made P25 paste. The light transmittance spectra of both IOT‐ETL, P25 mesoporous film and bare FTO were measured. As shown in Figure 2D, the thin IOT‐ETL not only remains excellent transmittance of short wavelength light but also promates the transmittance of long wavelength light, while the P25 mesoporous film demonstrates lower transmittance than bare FTO in the wavelength region from 300 to 600 nm. Due to the interesting property of antireflection, more light will arrive at the perovskite layer supported by IOT‐ETL.^19^ After the sequential reaction, no PbI_2_ diffraction peaks can be detected by XRD pattern (Figure S2B, Supporting Information), which indicates pores structure in IOT‐ETL film is suitable for the infiltration of CH_3_NH_3_I. We also investigated the morphology of the bottom in IOT‐ETL film obtained from the first spin‐coating of Ti(SO_4_)_2_ aqueous solution. Figure S3, Supporting Information, shows that the bottom is a dense and uniform film, which fully covers the whole FTO substrate, similar to the TiO_2_ compact film in previous reports.^20^

**Figure 2:**
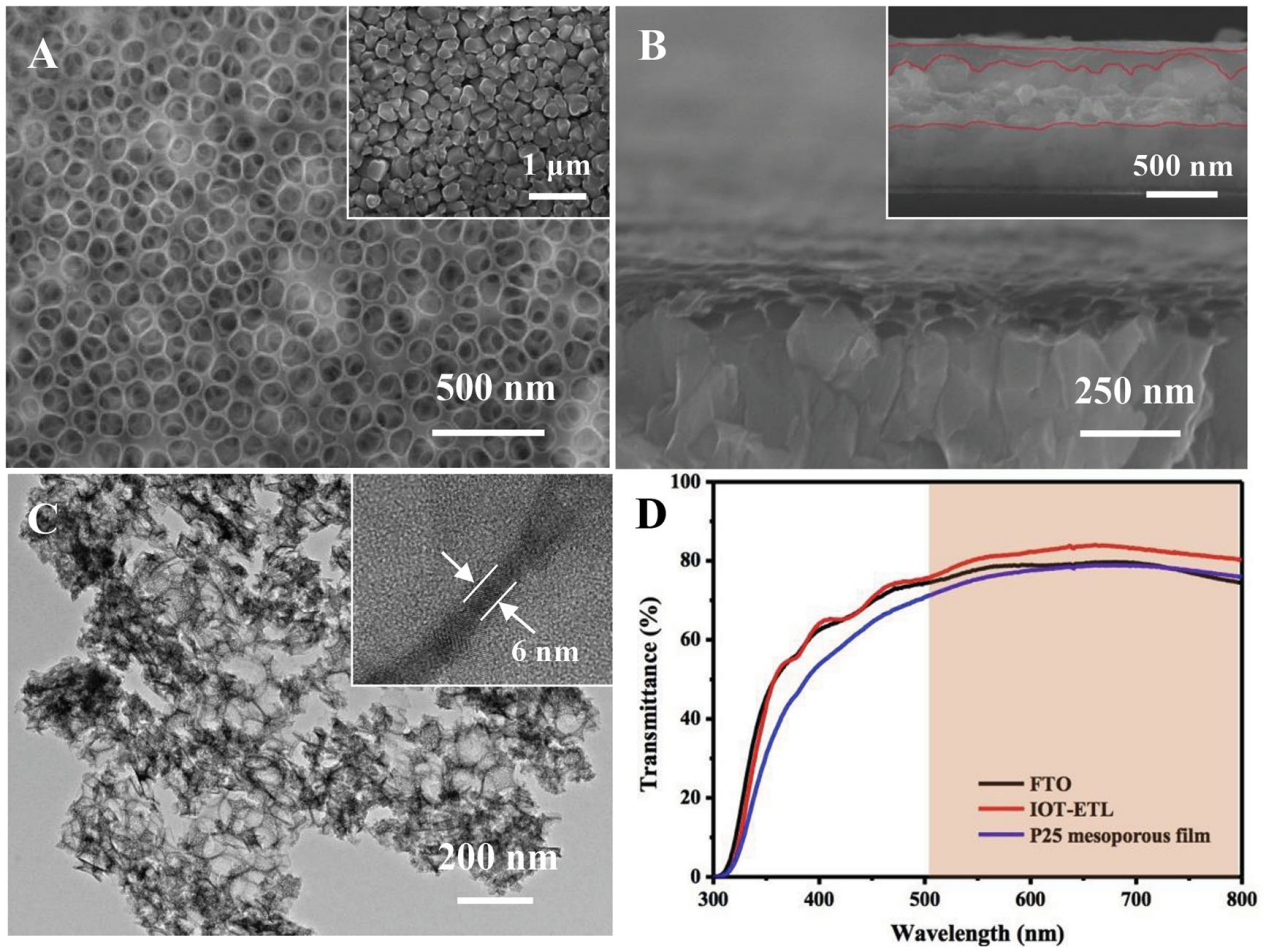
A) SEM image of the bare IOT‐ETL film, inset image is the CH_3_NH_3_PbI_3_ coated IOT‐ETL film, B) cross‐section SEM image of the bare IOT‐ETL film prepared under 4000 rpm, inset image is the cross‐section image of a device, C) TEM image of the bare IOT‐ETL, inset image is the high‐magnification TEM image of single wall of the bare IOT‐ETL film. D) Transmittance spectrum of bare FTO, TOT‐ETL, and P25 mesoporous film.

The current density (*J*)–voltage (*V*) characteristics of the pervoskite solar cells (4000 rpm 30 s for IOT‐ETL film preparation) with reverse scan under a simulated AM 1.5 G solar irradiation and in the dark are shown in **Figure** **3**A. Values of the short‐circuit current density (*J*_sc_), the open‐circuit potential (*V*_oc_) and the fill factor (*FF*) are 22 mA cm^−2^, 940 mV and 0.62, respectively, yielding a PCE of 12.78%. It is well known that the thickness of ETL films is closely related to the PCE of solar cells.^21^ To probe the effect of IOT‐ETL thickness on device performance, we further modify the thickness of IOT‐ETL films by changing the speed of spin‐coating with the same concentrate of precursor solution. From Figure 3B, the PCE value increases significantly with the decreasing of IOT‐ETL films thickness and reaches the highest value at 4000 rpm. A further decrease in the thickness has no influence on the improvement of device performance and this trend is related to declining charge generation efficiency, increased charge recombination and raised charge‐transport resistance.^22^ The average PCE value based on 20 samples of solar cells is 12.11% (Figure S4, Supporting Information), while the best‐performance solar cell using the scan direction from forward bias (FB) to short circuit (SC) with a scan rate of 0.15 V s^−1^ demonstrates a PCE value of 13.11% with 21.93 mA cm^−2^, 972.8 mV, and 0.61 for *J*_sc_, *V*_oc_, and *FF*, respectively (Figure 3C). To detect any hysteresis in the *J–V* curves, the IOT‐ETL films based perovskite solar cells were further measured using the forward and reverse scans with a scan rate of 0.15 V s^−1^. As a result, relatively obvious hysteresis are presented in Figure S5, Supporting Information, and larger pore size and smaller effective surface area compared to conventional P25 mesoporous film based devices are responsible for this phenomenon.^23^

**Figure 3:**
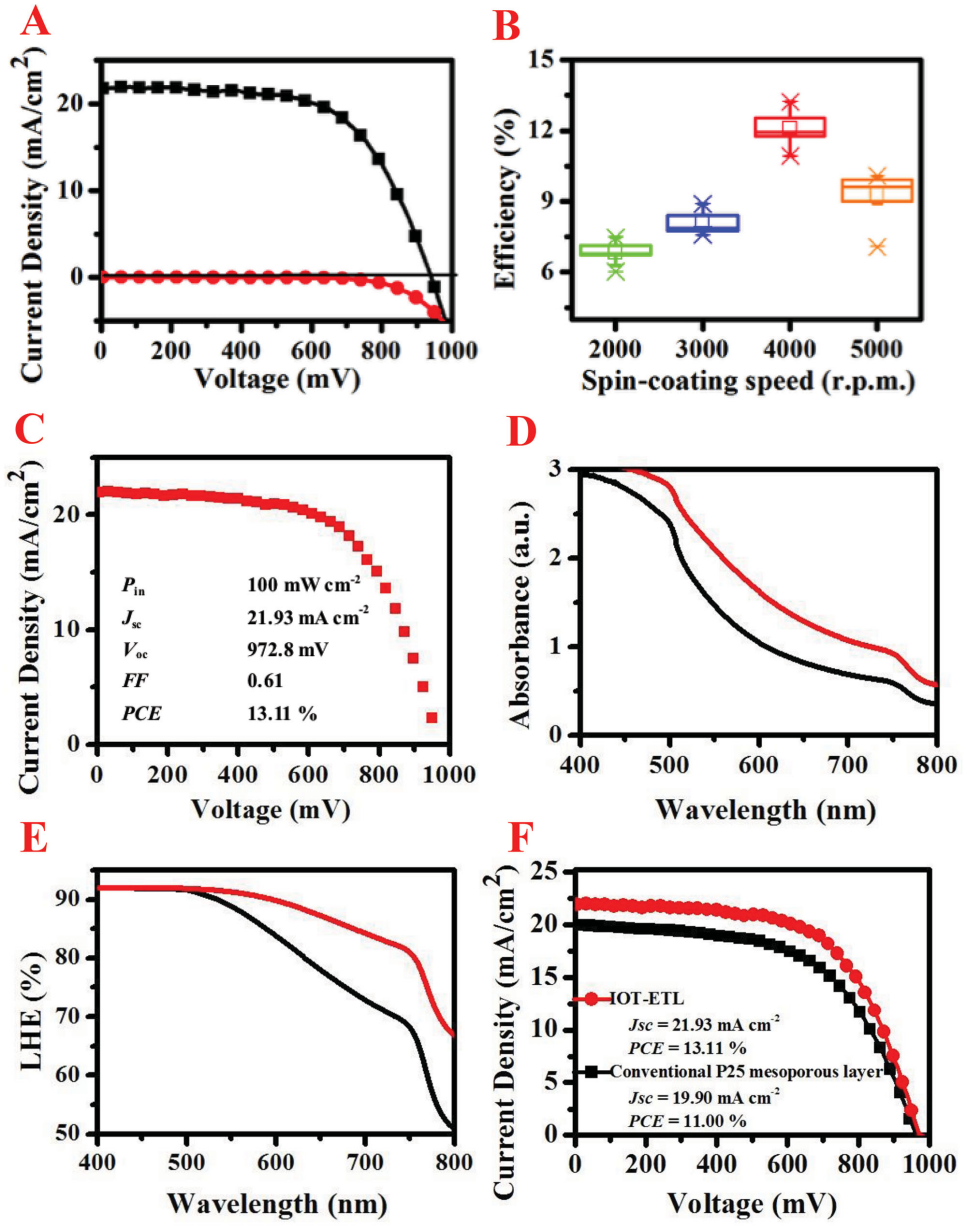
A) *J–V* curves for a IOT‐ETL based perovskite solar cell measured under a simulated AM 1.5 G solar irradiation (black cube) and in the dark (red circle), B) box charts of PCE for perovskite solar cells based on IOT‐ETL films obtained from various spin‐coating speed, C) *J–V* curves for the best‐performing solar cell (4000 rpm 30 s for IOT‐ETL film preparation) measured at a simulated AM 1.5 G solar irradiation, data of the power density of the incident light (*P*_in_), *J*_sc_, *V*_oc_, *FF* and PCE are also listed. D) Absorption spectrum of the perovskite‐coated IOT‐ETL film (red line) and conventional P25 mesoporous film (black line) on the FTO glass, E) LHE of the perovskite‐coated IOT‐ETL film (red line) and conventional P25 mesoporous film (black line) on the FTO glass, F) *J–V* curves for the IOT‐ETL and conventional P25 ETL based perovskite solar cell measured under a simulated AM 1.5 G solar irradiation.

Typical UV–vis absorption spectrum of CH_3_NH_3_PbI_3_ sensitized IOT‐ETL films and conventional P25 mesoporous films is presented in Figure 3D. The CH_3_NH_3_PbI_3_ sensitized electrodes show a panchromatic absorption of light with spectra extending from near‐UV to near infrared regions. The absorbance decreases gradually from 500 to 800 nm, which is consistent with results of previously reports.^14^ Moreover, CH_3_NH_3_PbI_3_ sensitized IOT‐ETL films demonstrate obviously higher absorption than that of conventional P25 mesoporous films, indicating the IOT‐ETL has unique optical property. LHE of the perovskite‐coated IOT‐ETL film on the FTO glass is shown in Figure 3E and the value of LHE is calculated by LHE = (1 – R)(1 – 10^−A^), where R and A represent reflectance and absorbance, respectively.^24^ It is demonstrated that the perovskite‐coated IOT‐ETL film shows obvious higher absorption from 500 to 800 nm than that of perovskite‐coated conventional P25 mesoporous film.^25^ Improved absorbance in long wavelength region can be attributed to the excellent light trapping ability of the perovskite‐coated IOT‐ETL film. Eventually, higher LHE will promote the generating of photoexcited electrons, enhance the *J*_sc_ and improve the PCE of pervoskite solar cells. *J–V* curves of two kinds of ETL films based devices are shown in Figure 3F and obvious increased *J*_sc_ from 19.9 mA cm^−2^ to 21.93 mA cm^−2^ leads to high‐performance of IOT‐ETL film based pervoskite solar cells, which confirms our inference. The external quantum efficiency (EQE) spectra of the best performing IOT‐ETL film based perovskite solar cell is close to full‐conversion as shown in Figure S6, Supporting Information. The integrated *J*_sc_ calculated from the EQE data is 20.59 mA cm^−2^, which agrees well with the measured *J*_sc_.

During the design process of IOT‐ETL, we compared the performance of IOT‐ETL films with and without bottom based perovskite solar cells. We found that the bottom of IOT‐ETL film fabricated by spin‐coating Ti(SO_4_)_2_ aqueous solution could prevent the direct contact between pervoskite and FTO glass and was critical to the improvement of the devices' PCE. **Figure** **4**A shows the *J–V* curves for perovskite solar cells based on different IOT‐ETL films. The bottomless IOT‐ETL film based solar cell exhibits a *J*_sc_ of 20.70 mA cm^−2^, a *V*_oc_ of 840 mV and a *FF* of 0.48, yielding a PCE of 8.28%. Obvious decrease of *V*_oc_ and *FF* is responsible for the low PCE, which attributes to the increased charge recombination without the bottom.^26^ In order to investigate the charge transport and recombination in the perovskite solar cells, the room temperature photoluminescence (PL) spectrum was measured with excitation at 600 nm. As shown in Figure 4B, the photoluminescence is quenched obviously for IOT‐ETL film with bottom, indicating the efficient exciton dissociation occurs at the interface between CH_3_NH_3_PbI_3_ and the bottom of IOT‐ETL and the electron–hole pair recombination is restricted.^27^ Further, we investigated the *J–V* characteristics under dark conditions for perovskite solar cells based on different IOT‐ETL films (Figure S4, Supporting Information). Might due to the charging‐discharging processed in perovskite or IOT‐ETL film, the lowest currents do not happen at zero bias.^28^ The bottomless IOT‐ETL film based solar cell demonstrates higher leakage current, indicating more charge recombination happens at the interface, which leads to the decrease of *V*_oc_ (schematic is shown in Figure 4C).^29^ In conclusion, the bottom in IOT‐ETL film has an equally important role with the compact layer in previous reports and the charge recombination occurring at the interface can be restricted effectively.^20^

**Figure 4:**
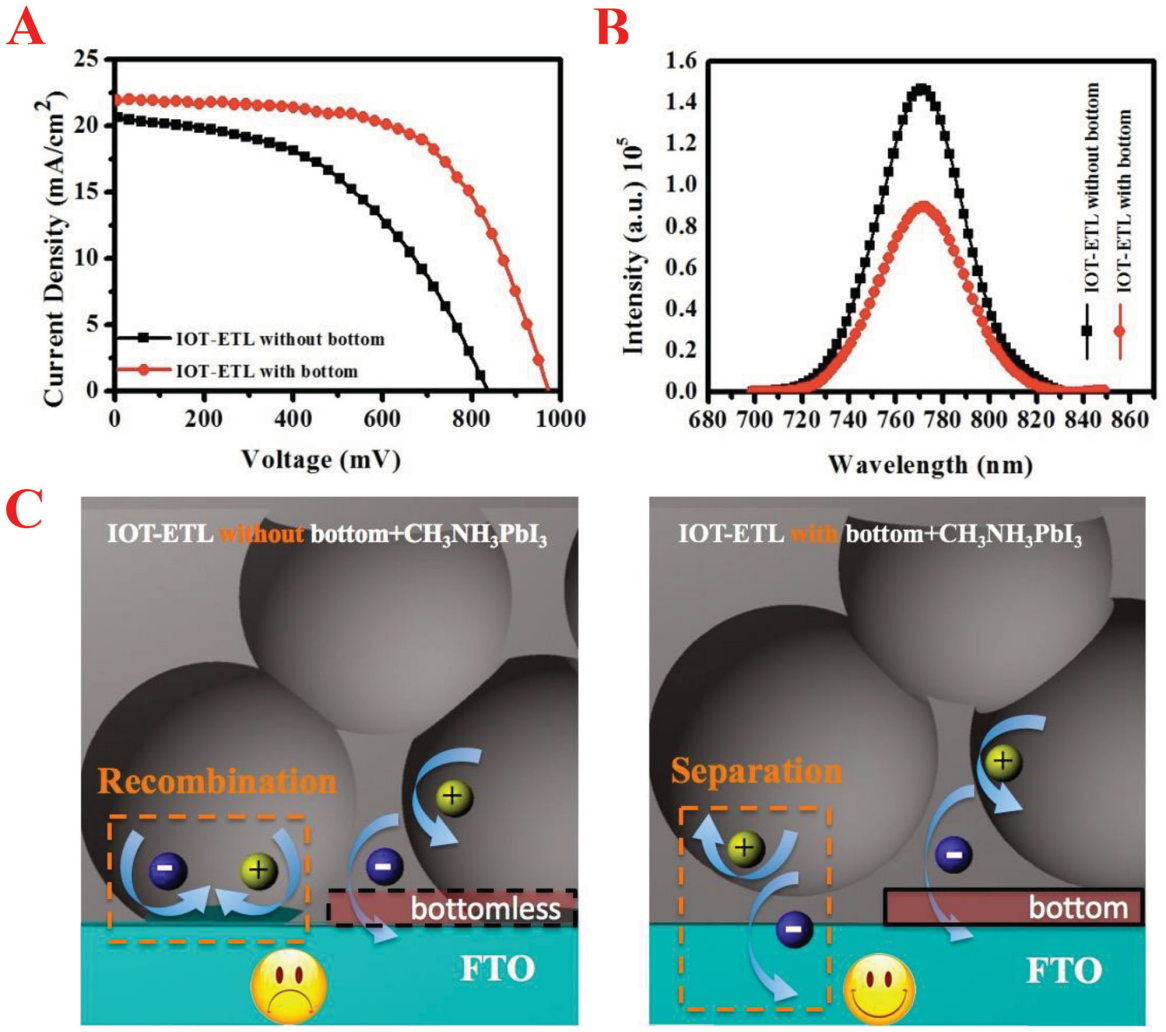
A) *J–V* curves for perovskite solar cells based on IOT‐ETL film with (red cricle) and without (black cube) bottom, measured under a simulated AM 1.5 G solar irradiation, B) room temperature photoluminescence (PL) spectrum of the perovskite coated IOT‐ETL film with (red cricle) and without (black cube) bottom (excitation at 600 nm), C) schematic illustrating the charge transfer and recombination in the perovskite coated IOT‐ETL film without (left) and with (right) bottom.

In summary, we have demonstrated a multifunctional inverse opal‐like TiO_2_ electron transport layer for efficient hybrid perovskite solar cells to replace the traditional compact layer and scaffold layer. The excellent light manipulation ability of IOT‐ETL film enhances the light harvesting efficiency, which improves the *J*_sc_ of solar cells. Moreover, the bottom in IOT‐ETL film significantly restricts the charge recombination, which leads to the increase of *V*_oc_. Eventually, the PCE of IOT‐ETL based solar cells has reached a high value of 13.11%. This multifunctional inverse opal‐like TiO_2_ based electron transport layer will be a promising photoelectrode selection in designing high‐performance and low‐cost pervoskite solar cells. More importantly, it will pave the way for introducing photonics structure in perovskite solar cells.

## Experimental Section

*Preparation of Self‐Made Polystyrene (PS) Nanospheres Emulsion*: Monodisperse PS nanospheres emulsion with the nanosphere size of 100 nm was prepared through a modified dispersion polymerization approach.^30^ For a typical preparation, 10 g of styrene (Sinopharm, AR), 1.5 g of polyvinylpyrrolidone (Alfa Aesar, M.W. 40 000) and 90 mL of deionized (DI) water were added into a 250 mL three‐neck round bottom flask, stirring for 30 min under the nitrogen atmosphere. After heated to 343 K, 10 mL of potassium persulfate solution (1 wt% in DI water) was injected into the three‐neck round bottom flask. The polymerization process was carried out for 24 h and the obtained PS emulsion was sealed for further use.

*Synthesis of Methylammonium Iodide (CH_3_NH_3_I)*: The CH_3_NH_3_I was prepared according to a reported method.^31^ The hydroiodic acid (30 mL, 57 wt% in water, J&K) and methylamine (27.86 mL, 40% in methanol, Sinopharm) were mixed and stirred at 273 K for 2 h. Crystallization of CH_3_NH_3_I was achieved using a rotary evaporator at 333 K for 1 h. The product was washed with diethyl ether three times and dried at 333 K in a vacuum oven for 12 h. The white coloured powder was formed indicating successful synthesis.

*Preparation of IOT‐ETL and Conventional P25 Mesoporous ETL on FTO*: The fluorine‐doped tin oxide (FTO) conducting glass substrate (NSG, 8 Ω/square) was patterned by etching with Zn powder and 2 m HCl diluted in DI water. The etched FTO substrate was then cleaned in an ultrasonic bath containing acetone for 20 min, ethanol for 20 min, rinsed with DI water and dried with clean dry air. The IOT‐ETL with 100 nm pore size was prepared by a simple two‐step spin coating titanium precursor solution (solution A and solution B) followed by sintering. Solution A was prepared by maxing 85 mg Ti(SO_4_)_2_ (Sinopharm, AR) with 1 mL DI water and solution B was made by mixing 85 mg Ti(SO_4_)_2_ and 1 mL the obtained PS emulsion followed by stiring and sonication. Solution A was spin‐coated on the FTO substrate at 4000 rpm for 30 s, which was heated at 398 K for 5 min. After cooling to room temperature, the solution B was spin‐coated on the obtained substrate at various spin speed for 30 s. Finally, the formed films were annealed at 773 K for 30 min with a heat ramp of 2 K min^−1^ to form inverse opal‐like TiO_2_ film. The IOT‐ETL film was immersed in 0.02 m aqueous TiCl_4_ (Sinopharm, AR) solution at 333 K for 40 min. After rinsing with DI water and drying, the film was heated at 773 K for 30 min. The bottomless IOT‐ETL film was prepared by the same method without the spin coating of solution A. Compact TiO_2_ blocking layer in conventional P25 mesoporous ETL was spin‐coated on the FTO substrate at 2000 rpm for 20 s using 0.15 m titanium diisopropoxide bis(acetylacetonate) (75 wt% in isopropanol, Aldrich) in 1‐butanol (99.8%, Aldrich) solution, followed by heated at 398 K for 5 min. After cooling to room temperature, the diluted P25 paste was spin‐coated on the compact TiO_2_ blocking layer at 5000 rpm for 30 s, where the pristine P25 paste was prepared by previous report^32^ and diluted in ethanol (0.13 g mL^−1^). The same thermal and TiCl_4_ treatment for IOT‐ETL film was further applied to P25 mesoporous ETL.

*Fabrication of Hybrid Perovskite Solar Cells*: The CH_3_NH_3_PbI_3_ was deposited on the ETL film using a two‐step method.^33^ The 1 m PbI_2_ solution was prepared by dissolving 462 mg PbI_2_ (99.9985%, Alfa Aesar) in 1 mL of *N,N*‐dimethylformamide (DMF, 99.9%, Alfa Aesar), stirring at 343 K for 12 h. The IOT‐ETL film was infiltrated with PbI_2_ by spin‐coating the 1 m PbI_2_ solution at 3000 rpm for 5 s and 6000 rpm for 5 s followed byheated at 373 K for 5 min. After cooling to room temperature, 100 μL of 6 mg mL^−1^ CH_3_NH_3_I solution in 2‐propanol (99.5%, Sigma‐Aldrich) was loaded on the PbI_2_‐coated film for 40 s, which was spun at 4000 rpm for 20 s and annealed at 373 K for 5 min. The HTM was then deposited by spin coating 2,2′,7,7′‐tetrakis(*N,N*‐di‐*p*‐methoxyphenylamine)‐9,9‐spirobifluorene (spiro‐MeOTAD) solution at 4000 rpm for 30 s. The spiro‐MeOTAD solution was prepared by dissolving 72.3 mg of spiro‐MeOTAD in 1 mL of chlorobenzene, to which 28.8 μL of 4‐tert‐butyl pyridine and 17.5 μL of lithium bis(trifluoromethanesulfonyl)imide (Li‐TFSI) solution (520 mg Li‐TSFI in 1 mL acetonitrile, Sigma‐Aldrich, 99.8%) were added. Finally, 110 nm Ag was thermally evaporated on the spiro‐MeOTAD‐coated film to form the back contact. The obtained solar cells were left in a desiccator for 72 h before tested. All the device fabrication processes were carried out under controlled atmospheric conditions and a humidity of <1%.

*Characterizations*: The morphology and structure of the IOT‐ETL films were characterized by field emission scanning electron microscopy (FESEM, HITACHI S4800) and high‐resolution transmission electron microscopy (HRTEM, JEOL JEM‐2010F, F20, 200 kV). The XRD spectra of the prepared IOT‐ETL films were measured using powder X‐ray diffraction (PXRD, Bruker D8 Advanced Diffractometer, Cu Kαradiation, 40 kV). The absorption spectra of the perovskite coated IOT‐ETL films were measured by using a Cary 500 UV–vis‐NIR Spectrophotometer. The photoluminescence measurement was acquired at room temperature using a UV–vis‐NIR fluorescene spectrophotometer (Fluorolog‐3‐P) with an excitation wavelength of 600 nm. The solar cells were illuminated using a solar light simulator (Oriel, 91160, AM 1.5 globe) and the power of the simulated light was calibrated to 100 mW cm^−2^ using a Newport Oriel PV reference cell system (model 91150 V). The *J–V* curves of solar cells were measured using a Keithley 2400 digital sourcemeter. Devices were masked with a metal aperture to define the active area of 0.0625 cm^2^. The EQE was measured using a Newport‐74125 system (Newport Instruments).

## Supporting information

As a service to our authors and readers, this journal provides supporting information supplied by the authors. Such materials are peer reviewed and may be re‐organized for online delivery, but are not copy‐edited or typeset. Technical support issues arising from supporting information (other than missing files) should be addressed to the authors.

Supplementary
