# Supplementary material for: Multifunctional Inverse Opal‐Like TiO2 Electron Transport Layer for Efficient Hybrid Perovskite Solar Cells
Source: Adv Sci (Weinh). 2015 Jun 17;2(9):1500105. doi: 10.1002/advs.201500105 (PMC5115377; doi:10.1002/advs.201500105)
Supplement: Supplementary file 1 — Supplementary [file ADVS-2-0l-s001.pdf]

Copyright WILEY-VCH Verlag GmbH & Co. KGaA, 69469 Weinheim, Germany, 2015.

ADVANCED SCIENCE
Open Access

Supporting Information

for *Adv. Sci.*, DOI: 10.1002/advs.201500105

Multifunctional Inverse Opal-Like TiO_2 Electron Transport Layer for Efficient Hybrid Perovskite Solar Cells

Xiao Chen, Shuang Yang, Yi Chu Zheng, Ying Chen, Yu Hou, Xiao Hua Yang,* and Hua Gui Yang*

Copyright WILEY-VCH Verlag GmbH & Co. KGaA, 69469 Weinheim, Germany, 2013.

Supporting Information

Multifunctional Inverse Opal-like TiO_2 Electron Transport Layer for Efficient Hybrid Perovskite Solar Cells

Xiao Chen, Shuang Yang, Yi Chu Zheng, Ying Chen, Yu Hou,Xiao Hua Yang, and Hua Gui Yang**

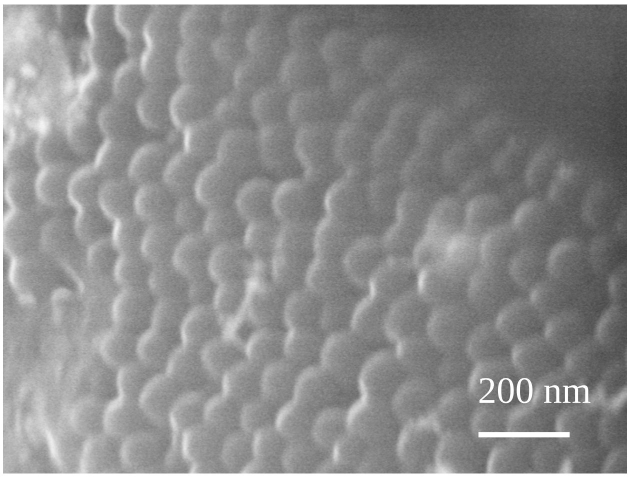

Figure S1. SEM image of the as-synthesized polystyrene (PS) nanospheres.

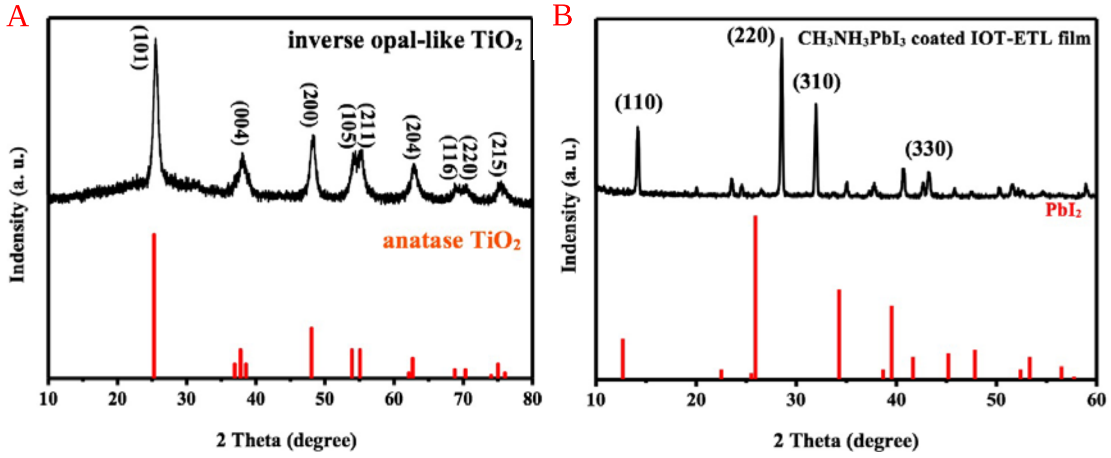

Figure S2. (A) XRD patterns of inverse opal-like TiO_2 powder, (B) X-ray diffraction patterns of $CH_3NH_3PbI_3$ coated IOT-ETL film (up) and pure PbI_2 (down).

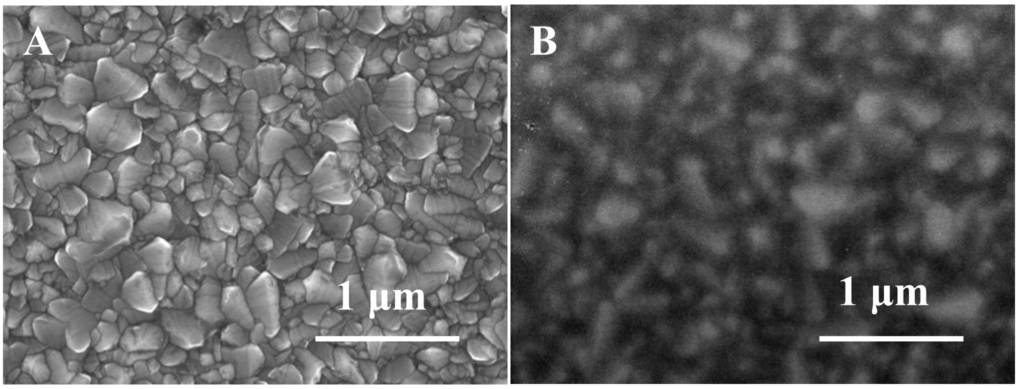

Figure S3. SEM images of bare FTO glass (A) and bottom of IOT-ETL film on FTO glass after sintered at 773 K for 30 min (B).

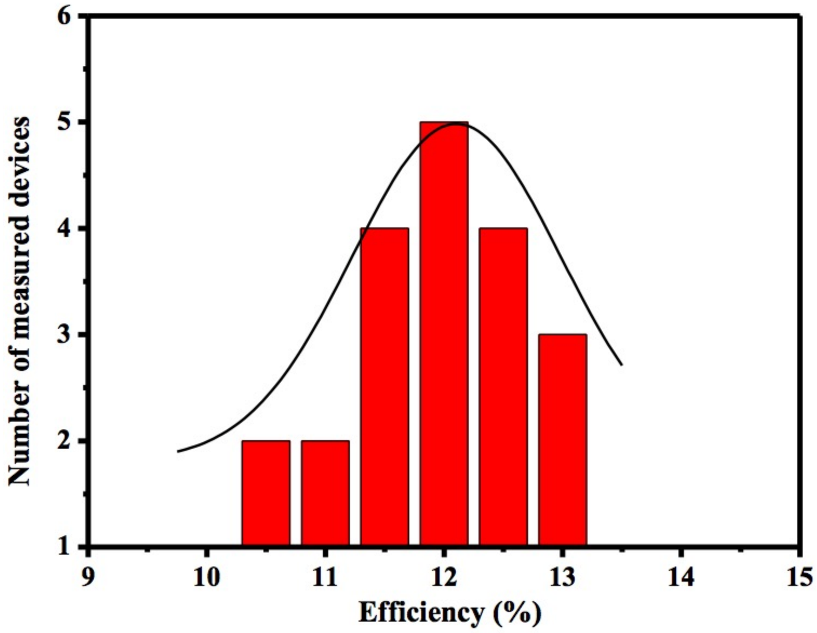

Figure S4. Histograms showing the variation in PCE for 20 individual perovskite solar cells based on IOT-ETL films.

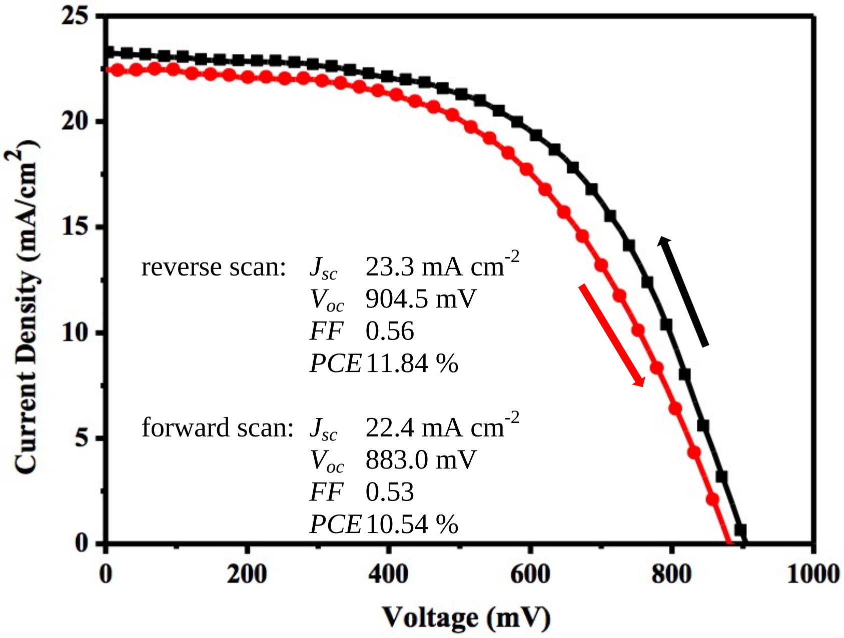

Figure S5. *J-V* curves for a IOT-ETL film based perovskite solar cell measured with different scanning directions.

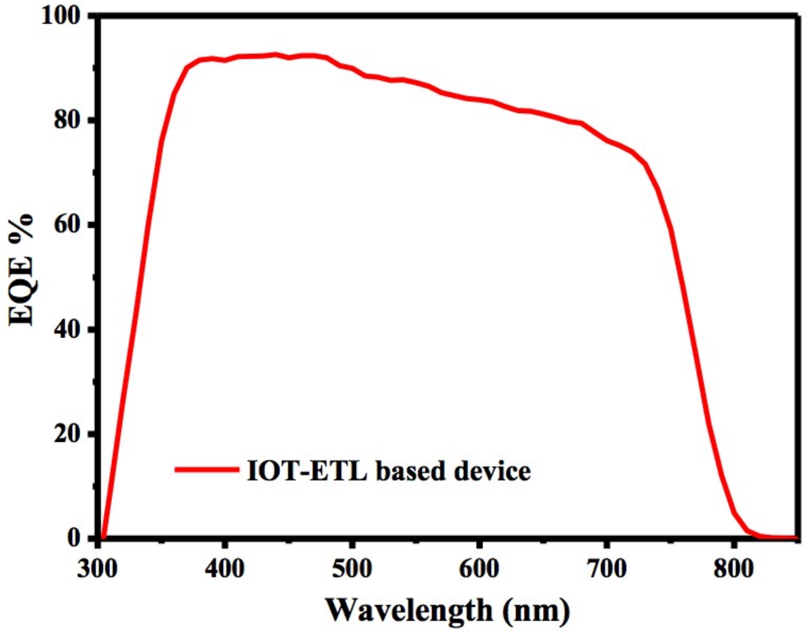

Figure S6. EQE spectra of the best performing IOT-ETL film based perovskite solar cell.

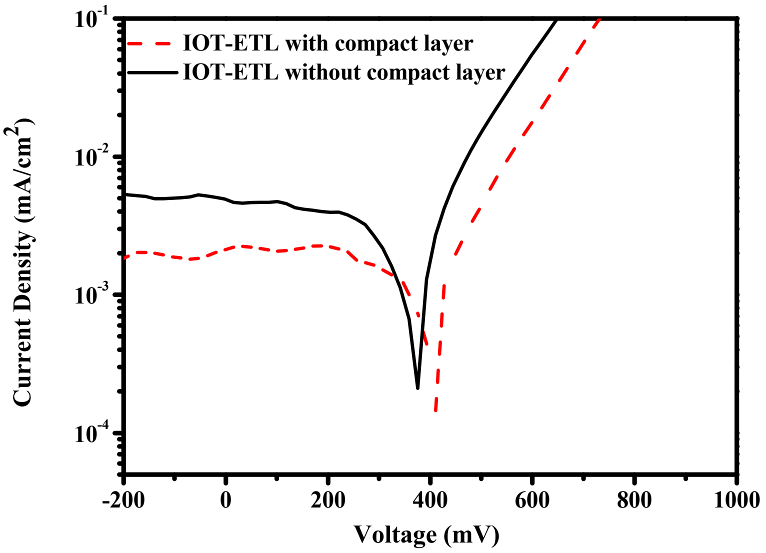

Figure S7. *J*-*V* curves for perovskite solar cells based on IOT-ETL films with and without bottom, measured under dark conditions.
